# Parents’ daily involvement in children’s math homework and activities during early elementary school

**DOI:** 10.1111/cdev.13774

**Published:** 2022-04-18

**Authors:** Jiawen Wu, Michael M. Barger, Dajung (Diana) Oh, Eva M. Pomerantz

**Affiliations:** ^1^ Department of Psychology University of Illinois at Urbana‐Champaign Champaign Illinois USA; ^2^ Department of Educational Psychology University of Georgia Athens Georgia USA

## Abstract

This research examined parents’ involvement in children’s math homework and activities. During 2017 to 2019, American parents (*N* = 483; 80% mothers; 67% white) of young elementary school children (*M*_age_ = 7.47 years; 50% girls) reported on their math helping self‐efficacy; they also reported on their involvement in children’s math homework and activities daily for 12 days. At this time and a year later, children’s math motivation and achievement were assessed. Parents’ involvement in homework (vs. activities) was more affectively negative (*d* = .34), particularly among parents low in self‐efficacy (*d* = .23). The more affectively negative parents’ involvement, particularly in homework, the poorer children’s later math motivation and achievement (*β*s = −.09 to .20).

**Abbreviation**
MLM: multilevel modeling

There is substantial evidence that throughout the school years children benefit when parents are involved in their learning (for reviews, see Barger et al., 2019; Pomerantz et al., 2012). Given the importance of math skills to societal advancement, investigators have increasingly focused on understanding how parents’ practices around math contribute to children’s math learning, with a major emphasis on parents’ involvement in math activities (e.g., playing number games and measuring while cooking) before children enter elementary school (for reviews, see Elliott & Bachman, 2018; Rowe et al., 2016). Once children enter elementary school, however, they regularly have math homework in which parents are often involved (e.g., Hyde et al., 2006). Math homework may be characterized by greater pressure and difficulty than are math activities. Consequently, math homework (vs. activities) may be a more challenging learning context for parents, which may undermine the quality of their involvement. Indeed, parents’ involvement in children’s learning during the elementary school years is less constructive (e.g., autonomy‐supportive) when parents feel pressured or children have difficulty (e.g., Grolnick et al., 2002; Wuyts et al., 2017).

Guided by *motivational models* in which parents foster children’s learning in general via the development of motivational resources (e.g., Grolnick et al., 1997; Pomerantz et al., 2012) and *cognitive models* in which parents foster children’s learning in math specifically via the development of math skills (e.g., Gunderson & Levine, 2011; Skwarchuk et al., 2014), the current research with children in early elementary school had three key aims. First, we examined the qualitative aspects (e.g., autonomy support and positive affect) of parents’ involvement in children’s math homework and activities to identify if one of these learning contexts fosters more constructive involvement. Second, given that parents can be anxious about math which appears to interfere with their involvement (DiStefano et al., 2020; Maloney et al., 2015), we examined whether parents’ feelings of efficacy in helping children with math serve as a resource for optimizing the qualitative aspects of their involvement in the math homework and activity contexts. Third, we evaluated whether the qualitative aspects of parents’ involvement in the math homework and activity contexts contribute to children’s math motivation and achievement.

## Parents’ involvement in math homework and activities

Many children experience heightened negative affect while doing homework (e.g., Leone & Richards, 1989), with parents sharing such affect when they assist with math (Else‐Quest et al., 2008). Parents’ negative affect may be intensified when children are struggling, which can be frustrating for parents but is often when they help with homework during elementary school (e.g., Pomerantz & Eaton, 2001; Silinskas et al., 2015). Indeed, during elementary and middle school, when children are frustrated with homework or doing poorly in school, parents’ involvement is more affectively negative (e.g., characterized by irritation) rather than positive (e.g., characterized by happiness) and controlling (e.g., intrusive and directive) rather than autonomy‐supportive (e.g., permitting initiative and choice; e.g., Dumont et al., 2014; Pomerantz et al., 2005; Silinskas et al., 2015). Although there has been relatively little research on parents’ involvement in children’s math homework specifically, the research to date indicates parents are often less constructively involved in such homework when children are struggling with math during adolescence (Silinskas & Kikas, 2019a, 2019b). Some parents’ involvement in math homework may be further undermined if they feel anxious about math, which is associated with affectively negative involvement with elementary school children (DiStefano et al., 2020).

In many families, in addition to the math homework assigned by teachers, parents, and children may regularly engage in formal and informal math activities (e.g., games and flashcards) at home. Research on such activities has been guided by cognitive models in which parents’ development of children’s math skills in the years before children enter elementary school is considered fundamental to children’s math learning (e.g., Gunderson & Levine, 2011; Skwarchuk et al., 2014). In this vein, there has been much attention to the types (e.g., constrained vs. unconstrained) of activities in which parents and children engage relevant to the development of math skills during these early years of children’s lives (e.g., McCormick et al., 2020; Skwarchuk et al., 2014). However, qualitative aspects of parents’ involvement (e.g., the extent to which it is controlling) were considered important in motivational models of parents’ role in children’s learning and examined in the homework context during the elementary and middle school years (e.g., Dumont et al., 2014; Pomerantz et al., 2005) have not received attention in the math activity context (for calls for such attention, see Elliott & Bachman, 2018; Rowe et al., 2016).

Similar to the math homework context, there may be variability in the qualitative aspects of parents’ involvement in the math activity context, but on average this context may elicit more autonomy‐supportive (vs. controlling) and affectively positive (vs. ‐negative) involvement than that of math homework. Unlike homework, activities are often not imposed on parents and children by an agent outside the home and do not have an evaluative component with a deadline. As such, math activities (vs. homework) may be characterized by less pressure. Drawing from self‐determination theory (Deci & Ryan, 1985), Grolnick and Apostoleris (2002) make the case that when parents feel pressured, they exert more control over children. Evidence from experimental and correlational studies with elementary school children and their parents is in line with the idea that pressure can lead parents’ involvement in children’s learning to be more controlling and less autonomy‐supportive (e.g., Grolnick et al., 2002; Wuyts et al., 2017), as well as more affectively negative (vs. positive; Grolnick, 2015). In addition, math activities may easily be adjusted to children’s math skills, either in terms of the choice of activities or changes made to activities as they unfold so children do not struggle too much, which may make constructive parenting easier for math activities than homework for which such flexibility is usually not an option.

## Parents’ self‐efficacy as a resource for parents’ involvement

Regardless of the context of parents’ involvement in children’s math learning, a key issue is that of what optimizes such involvement. Although parents likely bring multiple resources to their involvement in children’s math learning, of particular importance may be their feelings of efficacy in helping children with math—that is, the extent to which parents believe they are capable of supporting children in their math learning through their involvement (Hoover‐Dempsey & Sandler, 1997). In their model of the parent‐involvement process, Hoover‐Dempsey and Sandler (1997) draw from Bandura’s (1989) self‐efficacy theory to make the case that when parents feel efficacious in helping children succeed in school, they view themselves as capable of overcoming difficulties to do so. Consequently, they are able to sustain their involvement in children’s learning, even when they encounter challenge. There is substantial empirical evidence that the more parents feel efficacious in helping children succeed in school, the more involved they are in children’s learning in general (e.g., Green et al., 2007; Hoover‐Dempsey et al., 1992), as well as math specifically (Keating et al., 2021; O’Sullivan et al., 2014), during preschool, elementary school, and middle school.

Parents’ feelings of efficacy in supporting children’s math learning may also be important to the qualitative aspects of their involvement. Feelings of efficacy may allow parents to curb their negative affect when children are struggling with math as they believe that eventually they will be able to help children develop their skills. Such a belief may also mean that parents are less controlling as they may not feel the need to jump in and give children the answer before children start to struggle, which may be when parents worry the most about their effectiveness. Instead, parents may be more autonomy‐supportive in that they allow children to try and solve problems on their own, providing scaffolding as needed, which may be complex and thus require confidence on the part of parents. Although the link between parents’ self‐efficacy in supporting children’s learning and the quality of their involvement has not been examined, the tendency for math anxious parents to experience more negative affect in helping with children’s math homework (DiStefano et al., 2020) is suggestive of such a link. Parents’ self‐efficacy may be particularly important in the homework (vs. activity) context given that it may be more challenging for parents as it may be characterized by greater pressure and difficulty.

## Implications of parents’ involvement for children’s math motivation and achievement

Qualitative aspects of parents’ involvement in children’s learning appear to be important to the development of children’s motivation and achievement (for a review, see Pomerantz et al., 2012). In terms of math, when parents are affectively negative (vs. positive) in their interactions with children around math, they may not only transmit their negative affect to children, but also lead children to conclude math is not enjoyable and they lack the necessary abilities, which in turn may cause children to avoid challenging math, thereby undermining their math learning (e.g., Nolen‐Hoeksema et al., 1995; Pomerantz et al., 2005). Parents’ controlling (vs. autonomy‐supportive) involvement in math may dampen children’s feelings of autonomy and competence in this area, and thus their motivation and achievement in math (e.g., Grolnick & Ryan, 1989; Ng et al., 2004). The large body of research on qualitative aspects of parents’ involvement in children’s learning in general is in line with these ideas. For example, in the homework context, parents’ affectively negative (vs. positive) and controlling (vs. autonomy‐supportive) involvement predict dampened motivation and achievement over time among elementary and middle school children (e.g., Dumont et al., 2014; Pomerantz et al., 2005).

When it comes to parents’ involvement in children’s math activities, however, the picture may be more complex. Research on children prior to elementary school yields inconsistent results: Parents’ involvement in math activities sometimes has positive effects (e.g., Kleemans et al., 2012; Niklas & Schneider, 2014) but sometimes has no effects (e.g., DeFlorio & Beliakoff, 2015; Missall et al., 2015). This has led to the idea that the effects of such involvement depend in part on the extent to which activities support the development of relevant cognitive skills (e.g., LeFevre et al., 2009). For example, McCormick et al. (2020) found that parents’ involvement in math activities entailing unconstrained skills (i.e., limitless and acquired via varied experience rather than direct teaching), but not constrained skills (i.e., with a ceiling and acquired via direct teaching), were associated with children’s enhanced math achievement. Given the importance of relevant cognitive skills, qualitative aspects of parents’ involvement considered important in motivational models may only make a difference if the activities in which parents are involved support such skills, which homework may often do naturally as it is intended to allow children to practice skills relevant to what they are learning in school.

## Overview of the current research

To provide insight into parents’ involvement in children’s math homework and activities during early elementary school, we investigated three key issues in the current research. First, we compared the qualitative aspects of parents’ involvement in children’s math homework and activities. Second, we examined whether parents’ feelings of efficacy in helping children with math serve as a resource that optimizes the qualitative aspects of their involvement in the math homework and activity contexts. Third, we evaluated whether the qualitative aspects of parents’ involvement in these two contexts contribute to children’s math motivation and achievement. In examining these issues, we studied children in the early elementary school years (i.e., first and second grades) and their parents. At this time, math homework is often assigned on a regular basis and parents are more involved than in later years of schooling (Snyder et al., 2019). In addition, math activities started prior to children entering school may continue. The qualitative aspects of parents’ involvement in these early elementary school years may also be important in establishing the foundation for children’s later math motivation and achievement.

To examine parents’ involvement in the math homework and activity contexts, we used a 12‐day daily report to capture parents’ involvement in their ongoing day‐to‐day interactions with children in these two contexts. Most research on parents’ involvement in children’s learning has used retrospective reports in which parents provide information on the frequency or intensity of their practices in general (for some exceptions, see Pomerantz et al., 2005; Silinskas et al., 2015). Daily reports have some of the same drawbacks as retrospective reports (e.g., parents’ responses may be influenced by social desirability concerns) but provide more reliable and valid assessments (Bolger et al., 2003). For example, when asked at the end of the day about that day (vs. what they typically do), parents may be more likely to remember their interactions with children, thereby reporting accurately. In addition, daily reports allow for a more naturalistic assessment of parents’ practices in which time and energy constraints may interfere with acting in accordance with beliefs. In the current research, the use of daily reports provided the opportunity to capture not only the qualitative aspects of parents’ involvement in the math homework and activity contexts but also the frequency of their involvement in these contexts.

Parents’ daily reports were embedded in a longitudinal study. Parents completed the daily reports and reported on their feelings of efficacy in supporting children’s math learning when children were in first or second grade. At this time and a year later, two aspects of children’s math motivation were assessed. First, children reported on their math liking, an element of intrinsic motivation (Deci & Ryan, 1985) predictive of children’s math achievement over time during elementary school (e.g., Aunola et al., 2006). Second, an assessment was made of children’s preference for challenging math which is also predictive of children’s math achievement over time during elementary school (Sulik et al., 2020). In addition, children’s math achievement was also assessed at both time points. The inclusion of these measures at the time parents completed the daily checklist and a year later allowed the analyses to account for autoregression in predicting children’s math motivation and achievement over time from parents’ daily math involvement.

Given that the math homework context may be characterized by more pressure and difficulty than the math activity context, the first hypothesis was that parents’ involvement would be more controlling (vs. autonomy‐supportive) and more affectively negative (vs. positive) in the homework (vs. activity) context. To capture the broader landscape of the qualitative aspects of parents’ involvement in these two contexts, we also examined the frequency of children’s engagement in math homework and activities and parents’ involvement in these two math learning contexts. Our second hypothesis was that the more efficacious parents felt about helping children with math, the more involved they would be as in prior research (Keating et al., 2021; O’Sullivan et al., 2014) and the more their involvement would be autonomy‐supportive (vs. controlling) and affectively positive (vs. negative). The possibility that feelings of efficacy may be a more important resource in the more pressured and difficult context of math homework (vs. activities) was also explored. The third hypothesis was that as in research on parents’ involvement in children’s learning in general (for a review, see Pomerantz et al., 2012), the more autonomy‐supportive (vs. controlling) and affectively positive (vs. negative) parents’ involvement, the better children’s math motivation and achievement over time. These effects, however, may be stronger in the homework than activity context because homework may be more likely to support relevant cognitive skills. On the continuum of exploratory to confirmatory, these hypotheses lie somewhere in the middle in that they are based on prior theory and research to a large extent, but at the same time, most of the specific issues addressed by the hypotheses have not been directly studied in prior work.

## METHOD

### Participants

Participants were 483 children (50% girls) and one of their primary caregivers who were part of the Early Math Learning Project, which was carried out between 2015 and 2020 in the Midwest in a small urban area and surrounding areas, as well as a mid‐sized urban area. Participating primary caregivers (*M*_age_ = 38.17 years, *SD* = 6.59) were predominantly mothers (80%), with 17% being fathers, and 3% being other caretakers (e.g., grandmothers). Given that 97% of participating primary caregivers were mothers or fathers, we use the term “parents” throughout for simplicity. The majority (67%) of parents were white; 17% were Black, 8% were Asian, 5% were Latinx, and 4% were another ethnicity or more than one ethnicity. Parents’ highest level of educational attainment ranged from less than a college degree (29%) to an advanced graduate degree (38%). At the start of the project, children (*M*_age_ = 7.47 years, *SD* = 0.65) were in either first (55%) or second (45%) grade.

The sample on which this report is based is part of a larger sample of 614 parent‐child dyads who started the project 1–3 months prior to what is described here as Wave 1. Among the original sample, only dyads in which parents completed at least 1 day of the daily report over the 12‐day period were included in the sample described above. Parents who completed the daily report were more educated, *t*(609) = 5.09, *p* < .001, and more likely to be white, *χ* ^2^(11, *N* = 615) = 71.43, *p* < .001, than were parents who did not do so. Children of parents completing the daily report also scored higher on the math achievement test administered at the initial visit, *t*(614) = 6.87, *p* < .001.

### Procedure

Approximately 2 months after making the initial visit to the laboratory, parents received an online survey link every day for 12 days starting on a Monday. Each day, they reported on children’s engagement in math homework and activities along with their involvement in the homework and activities. Within approximately a month, parents and children visited the laboratory (Wave 1) for an assessment of parent’s efficacy in helping and children’s math motivation and achievement. Approximately a year later, parents and children visited the laboratory again (Wave 2) to complete assessments identical to those at the Wave 1 laboratory visit. At the initial visit (i.e., prior to Wave 1), half of the parents received math growth mindset information and half received math Common Core information. In each of these conditions, parents were either given math or non‐math activities to take home to do with children. Analyses including these experimental conditions as covariates yielded findings practically identical in size and significance to those reported in the Results section.

As a token of appreciation for their time and energy, parents received $55 for the Wave 1 visit and $95 for the Wave 2 visit; children received a small prize (e.g., rubber animal or eraser) at each visit. Among the parents who completed the daily reports, 89% completed both laboratory visits with their children. Although parents who completed both laboratory visits did not differ from parents completing only one visit, their children were more likely to be boys, *χ* ^2^(1, *N* = 483) = 5.14, *p* < .05, in second grade, *χ* ^2^(1, *N* = 483) = 10.75, *p* < .01, and score lower on the math achievement test administered at the initial visit (i.e., before Wave 1), *t*(480) = 2.67, *p* < .01. The procedures used in this project were approved by the University of Illinois Institutional Review Board (Protocol: The Early Math Learning Project, #16575).

### Measures

The means, standard deviations, and internal reliabilities for the parent and child measures are presented in Table 1; the correlations are presented in Table 2.

**TABLE 1:** Descriptives for parent and child measures

Variable	*n*	*M*	*SD*	Internal reliability	Correlation^a^
Parent measures					
Frequency of child engagement					
Homework	483	0.29	0.26	—	.00
Activity	483	0.26	0.26	—	
Frequency of parent involvement					
Homework	371	0.84	0.26	—	.04
Activity	352	0.83	0.28	—	
Autonomy support					
Homework	354	4.44	0.59	*r* = .48	.46***
Activity	330	4.38	0.63	*r* = .62	
Control					
Homework	353	1.37	0.66	*r* = .48	.48***
Activity	329	1.35	0.67	*r* = .62	
Positive affect					
Homework	352	3.96	0.84	*r* = .61	.50***
Activity	329	4.24	0.74	*r* = .66	
Negative affect					
Homework	346	1.35	0.58	*r* = .32	.55***
Activity	327	1.24	0.51	*r* = .26	
Efficacy in helping	469	8.33	1.55	*α* = .91	—
Child measures					
Math liking					
Wave 1	468	4.03	1.06	*α* = .82	.42***
Wave 2	437	3.96	1.06	*α* = .88	
Preference for math challenge					
Wave 1	468	0.47	0.24	—	.39***
Wave 2	435	0.52	0.22	—	
Math achievement					
Wave 1	466	475.76	22.18	—	.82***
Wave 2	437	493.49	22.28	—	

Note

The indexes for the frequency of child engagement can be interpreted as the proportion of days for which parents completed the daily reports in which children were engaged in the learning context. The indexes for the frequency of parent involvement can be interpreted as the proportion of days for which children engaged in the learning context in which parents were involved.

^a^ For the parent measures, correlations between the math homework and activity measures are presented. For the child measures, correlations between Wave 1 and 2 are presented.

*** *p* < .001.

**TABLE 2:** Correlations among Wave 1 variables

	1	2	3	4	5	6	7	8	9	10	11	12	13	14	15
1. Child engagement	—	.04	−.04	.05	.15**	−.12*	.15**	.07	.08	.01	−.06	−.13**	−.12**	.11*	−.07
2. Parent involvement	.06	—	−.01	−.03	.07	−.06	.03	−.02	−.04	−.04	−.03	−.04	−.07	−.04	−.05
3. Parent autonomy support	.01	−.01	—	−.11*	.27***	−.13*	.21***	.16**	.14*	.08	.10	.05	.12*	−.08	.04
4. Parent control	.19***	−.01	−.23***	—	−.03	.19**	−.18**	.00	−.03	−.18*	.03	.11*	−.03	−.15**	−.24***
5. Parent positive affect	.14*	.11*	.37***	−.09	—	−.42***	.40***	.10	.07	−.04	.07	−.07	−.07	−.03	−.05
6. Parent negative affect	.05	−.12*	−.20***	.25***	−.42***	—	−.43***	−.07	−.20***	−.11*	−.05	.01	.11*	−.13*	−.07
7. Parent efficacy	.11*	.05	.28***	−.13*	.40***	−.43***	—	.11*	.12**	.13**	.03	−.01	−.07	.22***	.09
8. Child liking	−.02	−.07	−.01	−.01	.10	−.07	.11*	—	.64***	.05	.05	−.02	−.01	.00	−.02
9. Child challenge	−.09	−.06	.07	−.05	.07	−.20***	.12**	.64***	—	.20***	.04	.07	−.03	.05	.07
10. Child achievement	−.07	−.16**	.06	−.16**	−.04	−.11*	.13**	.05	.20***	—	−.03	.53***	−.08	.32***	.13**
11. Child gender	.02	−.05	.13*	−.05	.07	−.05	.03	.05	.04	−.03	—	.06	.06	−.02	.02
12. Child grade	.02	−.18**	.01	−.05	−.07	.01	−.01	−.02	.07	.53***	.06	—	.02	.00	.01
12. Parent gender	−.06	.04	−.03	.01	−.07	.11*	−.07	−.01	−.03	−.08	.06	.02	—	−.12**	.03
14. Parent education	−.08	−.05	.10	−.11*	−.03	−.13*	.22***	.00	.05	.32***	−.02	.00	−.12**	—	.21***
15. Number of daily reports	−.31**	−.11*	.12*	−.26***	−.05	−.07	.09	−.02	.07	.13**	.02	.01	.03	.21***	—

Note

The correlations in the lower triangle are for the summary indexes (i.e., across days parents completed the daily reports or children were engaged in the context) for the math homework context measures; the correlations in the upper triangle are for the summary indexes for the math activity context measures. For child gender, −1 = boys and 1 = girls; for child grade, −1 = first grade and 1 = second grade; for parent gender, −1 = fathers, 1 = mothers; for parents’ education, −1 = less than a bachelor's degree, 0 = a bachelor's degree or equivalent, and 1 = an advanced graduate degree.

* *p* < .05

** *p* < .01

*** *p* < .001.

#### Parent daily assessments

Almost all parents completed the daily reports via an electronic device (e.g., smartphone, tablet, or computer), with a few doing so over the phone with a trained research assistant asking each question and recording parents’ responses. On average, parents completed the daily report for 9.26 days (*SD* = 3.34), with 36% completing the report for all 12 days and 5% completing it for only 1 day (for information on the samples for the different daily measures, see Figure S1).

##### Frequency of child math engagement

Each day of the daily report, parents indicated whether children had math homework (0 = *no homework*, 1 = *homework*) and then if they engaged in any other math activities (0 = *no activity*, 1 = *activity*) with examples of such activities (i.e., telling time, measuring things around the house, measuring while cooking, math flashcards, math board or card games, or math computer games) provided. If parents indicated children engaged in math activities, they were asked to indicate what kind of activities from a list provided (for the activities and their frequency, see Table S1). Seventy‐seven percent of parents indicated children had homework on at least 1 day (range = 0–10 days) during the 12‐day period; 73% indicated children did a math activity on at least 1 day (range = 0–10 days). Slightly over half (57%) of parents reported engagement in both math homework and activities at least once over the 12‐day period. We calculated summary indexes of the frequency of children's math *homework engagement* and math *activity engagement* by taking the means for each math context across the days for which parents completed the daily checklist, which given the zero‐ versus one‐coding may be interpreted as the proportion of days for which parents completed the daily reports that children were engaged in the math learning contexts.

##### Frequency of parent math involvement

If parents indicated children engaged in math homework or activities, they were then asked if they interacted with children around the homework or activities (0 = *not involved*, 1 = *involved*). Among families in which children had math homework, 95% of parents said they were involved on at least 1 day (range = 0–9 days); among families in which children had math activities, 94% of parents indicated they were involved on at least 1 day (range = 0–10 days). We calculated summary indexes of the frequency of parent’s math *homework involvement* and *activity involvement* by taking the mean of each type of involvement across the days, children were engaged in the math context, which given the zero‐ versus one‐coding may be interpreted as the proportion of days children were engaged in the math learning context that parents were involved.

##### Qualitative aspects of parent math involvement

On days parents were involved in children’s math homework or activities, they reported on the qualitative aspects of their involvement in the context. The scales for each aspect were kept brief to maintain parents’ participation throughout the full 12 days of the daily report. Parents’ *autonomy*‐*supportive* (e.g., “I permitted or encouraged my child to try to figure out how to solve problems on his/her math homework [activity] in his/her own way.”) and *controlling* (e.g., “I insisted my child do things my way when it came to doing his/her math homework [activity].”) involvement were each assessed with two items adapted from Cheung et al. (2016) scales of these types of parenting in the academic context which were based on more general measures of autonomy‐supportive and controlling parenting (e.g., Barber, 1996; Steinberg et al., 1992; Wang et al., 2007). For both autonomy‐supportive and controlling involvement, parents indicated on a 5‐point Likert scale the extent (1 = *not at all true*, 5 = *very true*) to which they used it in the context that day. Autonomy support and control appear to represent separate, albeit related, dimensions of the quality of parents’ involvement as they tend to be only modestly inversely correlated (e.g., Cheung & Pomerantz, 2011; Silk et al., 2003) as was the case in the current research (*r*s = −.23 for homework and −.11 for activities, *p*s < .05). Thus, the mean of the two items comprising the scale for each type of involvement was taken for each math context for each day, such that higher scores indicate parents were more autonomy‐supportive or controlling in the context for that day. A summary index was created by taking the mean across the days for which parents were involved.

Building on Pomerantz et al.’s (2005) and Silinskas et al.’s (2015) measures, we assessed parents’ affectively positive and negative involvement in math homework and activities. For each context, *affectively positive involvement* was assessed with two items (i.e., happy and content) as was *affectively negative involvement* (i.e., irritated and anxious). Parents indicated on a 5‐point Likert scale how much they experienced each emotion (1 = *did not feel this way at all*, 5 = *very much felt this way*) while interacting with children in the context that day. Parents’ affectively positive and negative involvement appear to represent distinct, albeit related, dimensions of parents’ involvement as they are only modestly inversely correlated (e.g., Pomerantz et al., 2005; Silinskas et al., 2015) as was the case in the current research (*r*s = −.42 for homework and −.21 for activities, *p*s < .01). Thus, the mean of the two items for each type of involvement was taken for each math context for each day, such that higher scores reflect more affectively positive or negative involvement in the context for that day. A summary index was created by taking the mean across the days for which parents were involved.

#### Parent self‐efficacy in helping with math

Parents’ feelings of efficacy in helping children with math were assessed by adapting Hoover‐Dempsey and Sandler’s (1997) self‐efficacy for helping children succeed at school to the math arena. Parents indicated on a 10‐point Likert scale the extent to which six items about their feelings of efficacy in helping children with math (e.g., “I feel confident in my ability to help my child develop his/her math skills.” “I know how to help my child do well in math.”) were true of them (1 = *not at all true*, 10 = *very true*). Four of these overlapped with the four items comprising O’Sullivan et al.’s (2014) adaptation of Hoover‐Dempsey and Sandler’s (1995) scale to the math arena. The mean of the six items was taken, with higher numbers reflecting greater feelings of efficacy.

#### Child math motivation and achievement

At both the Wave 1 and 2 laboratory visit, children’s math motivation and achievement were assessed.

##### Math liking

Children’s math liking was assessed with three items. One item (“How much do you like doing math?”) has been used in prior measures of math liking with elementary school children (e.g., Aunola et al., 2006; Eccles et al., 1993); two similar items (e.g., “How much do you enjoy doing math?”) were created for the current research. Children responded to each using a 5‐point Likert scale with five circles that became larger from left to right. A trained research assistant explained to children that the smallest circle meant *not at all* with the next circle *being a little true*, the third being *somewhat true*, the fourth being *pretty much true*, and the largest being *a lot true*. Children went through an example (i.e., “I think vanilla is better than chocolate ice cream.”) with the research assistant before using the scale for their math liking. The mean of the three items was taken, with higher numbers reflecting more math liking.

##### Math challenge preference

We adapted Yeager et al.’s (2016) Make‐a‐Math‐Worksheet measure of challenge preference for college students to be used with the young children in the current study. Children were told they would be working on a math worksheet they would make themselves by choosing the problems to be included on it. There were four types of math problems (i.e., addition, subtraction, time, and coins). For each type, children chose three problems from a set of three easy and three hard problems. The worksheet had three empty boxes for each type (e.g., addition) of problem. Children placed a laminated square, which was labeled with words and colors (i.e., “easy” squares were blue and “hard” squares were yellow) as well as verbally by the research assistant, in each box. For each type of problem, children could choose from zero to three hard problems, with the remaining being easy problems. A preference for challenge index was created by calculating the proportion of hard problems out of the total of 12 problems, with higher numbers indicating a greater preference for difficult (vs. easy) math.

##### Child math achievement

Children’s math achievement was assessed with the Applied Problems subtest of the Woodcock–Johnson III Tests of Achievement (Woodcock et al., 2001). This test assesses the application of math knowledge, calculation skills, and quantitative reasoning. The raw scores were transformed into Rasch‐scaled scores with equal intervals yielding W scores, which are recommended as they take into account children’s grade in school and are suitable for examining individual growth over time (Woodcock et al., 2001).

## RESULTS

We conducted three central sets of analyses. First, we used multilevel modeling (MLM) to compare the qualitative aspects of parents’ involvement in the math homework and activity context. To elucidate the broader landscape of parents’ involvement we also examined the frequency of children’s engagement and parent’s involvement in the two contexts with MLM. Second, we used MLM to investigate the link between parents’ self‐efficacy in helping with math and parents’ involvement in the math homework and activity contexts. Third, we used multiple regression to identify if the qualitative aspects of parents’ involvement contribute to children’s math motivation and achievement over time.

### Comparison of the math homework and activity context

The first set of analyses focused on whether the qualitative aspects of parents’ involvement in math homework and activities differ. To capture the broader landscape, we also examined the frequency of children’s engagement and parents’ involvement in both contexts. We took a multilevel analytic approach as it allows for the examination of individual‐level differences (i.e., between math homework and activities) without violating assumptions of independence given the nested structure of the data (i.e., two learning contexts nested within multiple days nested within families). Such an analytic approach is unbiased in the presence of an unequal number of events, which is important in the current research given the variation in not only the number of days parents completed the daily reports, but also the number of days children were engaged and parents were involved in math homework and activities. In estimating the intercepts, this approach includes families who engaged at least one type of math learning context at least once over the 12 days; in estimating the context slopes, this approach includes families who engaged in both types at least once over the 12 days (for the MLM equations and information on the sample sizes for each daily report variable, see Supporting Information). The MLMs were conducted using the *lme4* (v. 1.1‐7; Bates et al., 2014) package in R (v 3.6.1). Degrees of freedom was computed using the Satterthwaite (1946) method.

Logistic MLM was carried out on the frequency of children’s engagement and parents’ involvement, which were both dichotomous (0 = *no occurrence*, 1 = *occurrence*) at the daily level. We began with unconditional models in which children’s engagement, for example, was predicted from the math learning context (−1 = *homework*, 1 = *activity*) at Level 1 nested within days (Level 2) nested within families (Level 3). As shown in Table 3, there was not an effect of the math learning context on the frequency of children’s engagement, Wald‐*z* = −1.23, *p* = .22, or parents’ involvement, Wald‐*z* = −0.48, *p* = .63. Children were engaged in math homework to the same extent as math activities. Parents reported children as engaged in math homework and activities about a quarter of the time they completed the daily reports; once children were engaged, parents were involved on average slightly over 80% of the time in each of the two contexts (see Table 1). There was variability in the effect of context between dyads for both children’s engagement, *σ* = .14, and parents’ involvement, *σ* = .11.

**TABLE 3:** Logistic multilinear models predicting the frequency of child engagement and parent involvement at Wave 1

Predictors	Child engagement frequency		Parent involvement frequency	
	*b* (*SE*)	Cohen’s *d*	*b* (*SE*)	Cohen’s *d*
Unconditional model				
Intercept	−1.15 (.04)***	—	1.86 (.09)***	—
Context slope	−0.03 (.02)	−.03	−0.03 (.06)	−.03
Conditional model				
Intercept	−2.00 (.24)***	—	0.62 (.51)	—
Parent education	0.04 (.05)	.05	−0.13 (.11)	−.11
Parent gender	−0.11 (.05)*	−.20	0.01 (.11)	.02
Child gender	−0.05 (.04)	−.10	−0.10 (.08)	−.11
Child grade	−0.05 (.04)	−.12	−0.25 (.09)**	−.30
Parent efficacy	0.11 (.03)***	.35	0.16 (.06)**	.28
Context slope	−0.16 (.16)	−.02	0.13 (.42)	.01
Parent education	0.21 (.03)***	.14	0.06 (.09)	.03
Parent gender	−0.03 (.03)	−.02	−0.12 (.09)	−.07
Child gender	−0.07 (.03)**	−.05	−0.07 (.07)	−.06
Child grade	−0.16 (.03)***	−.14	0.13 (.07)	.10
Parent efficacy	0.01 (.02)	.01	−0.01 (.05)	−.01

Note

The unconditional models include 18 dyads for child engagement and 16 dyads for parent involvement not included in the conditional models because these dyads were missing at least one of the variables necessary for the conditional model. The effect sizes (Cohen’s *d*s) were calculated following Westfall et al.’s (2014) guidelines using the *EMAtools* (Kleiman, 2017) package in R (v 3.6.1). For parents’ education, −1 = less than a bachelor’s degree or less, 0 = a bachelor’s degree or equivalent, and 1 = an advanced graduate degree; for parent gender, −1 = fathers, 1 = mothers; for child gender, −1 = boys and 1 = girls; for child grade, −1 = first grade and 1 = second grade, for context, −1 = homework and 1 = activity.

* *p* < .05

** *p* < .01

*** *p* < .001.

Comparisons of the qualitative aspects of parents’ involvement in the homework and activity contexts were made with linear MLM given that such aspects were continuous (e.g., 1 = *not at all true*, 5 = *very true*). Similar to the logistic MLMs, each qualitative aspect (i.e., autonomy support, control, positive affect, and negative affect) was predicted from the math learning context at Level 1 nested within days (Level 2) nested within families (Level 3). Surprisingly, as shown in the unconditional models in Table 4, parents’ autonomy support was higher for children’s math homework than activities, *t*(941) = −2.33, *p* < .05, but their control did not differ in the two contexts, *t*(839) = 0.34, *p* = .73. As anticipated, parents were less affectively positive and more affectively negative when involved in children’s math homework than activities, |*t*|(877)s > 3.71, *p*s < .001. For all four types of involvement, there was at least some variability in the effect of context, with control having the least variability, *σ* = .08, autonomy support, *σ* = .11, and negative affect, *σ* = .13, having slightly more variability, and positive affect, *σ* = .22, having the most variability.

**TABLE 4:** Multilinear models predicting the qualitative aspects of parent involvement at Wave 1

Predictors	Autonomy support		Control		Positive affect		Negative affect	
	*b* (*SE*)	Cohen’s *d*	*b* (*SE*)	Cohen’s *d*	*b* (*SE*)	Cohen’s *d*	*b* (*SE*)	Cohen’s *d*
Unconditional model								
Intercept	4.41 (.03)***	—	1.35 (.03)***	—	4.09 (.03)***	—	1.30 (.02)***	—
Context slope	−0.03 (.01)*	−.15	0.00 (.01)	.02	0.11 (.02)***	.45	−0.04 (.01)***	−.34
Conditional model								
Intercept	3.54 (.15)***	—	1.95 (.16)***	—	2.51 (.20)***	—	2.32 (.13)***	—
Parent education	−0.03 (.03)	−.10	−0.08 (.03)*	−.27	−0.14 (.04)***	−.33	−0.02 (.03)	−.07
Parent gender	0.04 (.03)	.12	−0.03 (.03)	−.09	0.01 (.04)	.03	0.03 (.03)	.12
Child gender	0.06 (.02)*	.24	0.02 (.03)	.11	0.03 (.03)	.09	0.00 (.02)	−.02
Child grade	0.02 (.02)	.06	0.01 (.03)	.03	−0.04 (.03)	−.14	0.01 (.02)	.05
Parent efficacy	0.10 (.02)***	.56	−0.07 (.02)***	−.39	0.19 (.02)***	.78	−0.12 (.02)***	−.83
Context slope	−0.11 (.09)	−.07	0.23 (.09)**	.17	0.34 (.11)**	.18	−0.23 (.07)***	−.25
Parent education	−0.06 (.02)**	−.19	0.01 (.02)	.04	−0.01 (.02)	−.02	−0.02 (.01)	−.11
Parent gender	0.03 (.02)	.13	−0.01 (.02)	−.03	0.09 (.02)***	.31	−0.02 (.01)	−.15
Child gender	0.01 (.01)	.03	0.02 (.01)	.11	−0.01 (.02)	−.03	0.00 (.01)	.01
Child grade	0.01 (.01)	.06	0.03 (.01)*	.14	0.03 (.02)	.12	−0.01 (.01)	−.09
Parent efficacy	0.01 (.01)	.05	−0.02 (.01)*	−.16	−0.03 (.01)*	−.15	0.02 (.01)**	.23

Note

The unconditional model includes 15 dyads not included in the conditional model because these dyads were missing at least one of the variables necessary for the conditional model. The effect sizes (Cohen’s *d*s) were calculated followed Westfall et al. (2014)’s suggestion using *EMAtools* (Kleiman, 2017) package in R (v 3.6.1). For parents’ education, −1 = less than a bachelor’s degree, 0 = a bachelor’s degree or equivalent, and 1 = an advanced graduate degree; for parent gender, −1 = fathers, 1 = mothers; for child gender, −1 = boys and 1 = girls; for child grade, −1 = first grade and 1 = second grade, for context, −1 = homework and 1 = activity.

* *p* < .05

** *p* < .01

*** *p* < .001.

### Examination of associations with parents’ self‐efficacy

To examine if parents’ feelings of efficacy in helping children with math have the potential to serve as a resource for parents in their involvement in children’s math learning, we added to the unconditional models reported above, parent’s self‐efficacy as a grand‐centered predictor at the family level (Level 3) of both the intercept and the context slope. We also included four potential demographic confounds at the family level in predicting the intercept and slope: (1) parents’ educational attainment, (2) parents’ gender, (3) children’s gender, and (4) children’s grade in school. Because 14 parents were missing the self‐efficacy measures and four were missing educational attainment information, this conditional model was based on a slightly smaller sample, which was used in nested comparisons of the conditional and unconditional models. These comparisons indicated that the conditional models provided a better fit, Δ*χ^2^* s(10) > 21.91, *p*s < .01. To ensure this reflected the specific contribution of parents’ self‐efficacy in helping and not the demographic covariates, we conducted nested comparisons of models with and without parents’ self‐efficacy. Results indicated that the inclusion of parents’ self‐efficacy significantly improved model fit in comparison to the models with only the demographic covariates, Δ*χ^2^* s(2) > 8.72, *p*s < .05, suggesting that parents’ self‐efficacy is important in understanding variation in children’s engagement and parents’ involvement.

As shown in Table 3, the more parents felt efficacious in helping children with math, the more children engaged in math homework and activities, Wald‐*z* = 3.79, *p* < .001, and the more parents themselves were involved in t contexts, Wald‐*z* = 2.72, *p* < .01. Parents’ feelings of efficacy were also predictive of more constructive involvement in that the more efficacious parents felt, the more they were autonomy‐supportive and affectively positive and the less they were controlling and affectively negative (see Table 4), |*t|*s > 3.56, *p*s < .001. Notably, these effects of parents’ self‐efficacy were evident over and above the demographic covariates, which were also predictive on their own in three cases: (1) parents were more involved with children in first than second grade, Wald‐*z* = −2.91, *p* < .01, (2) more educated parents were less controlling, *t*(312) = −2.36, *p* < .05, and less affectively positive, *t*(417) = −3.41, *p* < .001, and (3) parents were more autonomy‐supportive with girls than boys, *t*(381) = 2.37, *p* < .05.

**FIGURE 1:**
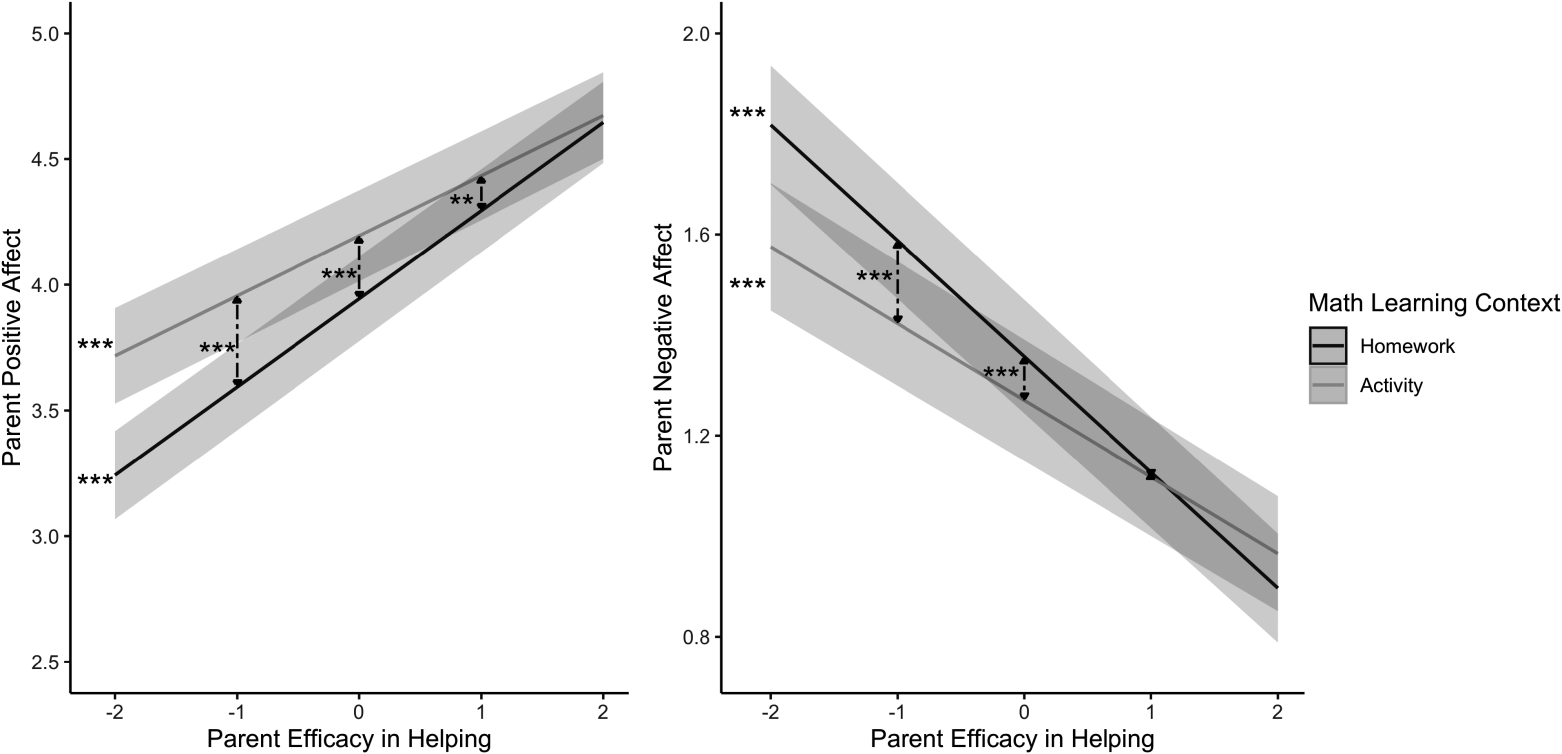
Interaction between parent efficacy in helping and the math learning context in predicting parent positive and negative affect at Wave 1. *Note*: Parent efficacy in helping was standardized across participants, such that −1 is 1 *SD* below the mean, 0 is the mean, and 1 is 1 *SD* above the mean

Although the effects of parents’ self‐efficacy in helping on the frequency of children’s engagement and parents’ involvement did not vary with the learning context, |Wald‐*z*|s < 0.54, *p*s > .59, its effects on the qualitative aspects of parents’ involvement did (see Table 4). Indeed, the effect of parents’ self‐efficacy on parents’ positive and negative affect varied by context, |*t*|s > 2.44, *p*s < .05: As shown in Figure 1, decomposition of the effects of parents’ self‐efficacy on the context slope for positive and negative affect indicated that although parents’ self‐efficacy in helping predicted more positive and less negative affect in both the homework (*β*s = .34 for positive affect and −.23 for negative affect, *p*s < .01) and activity (*β*s = .24 for positive affect and −.15 for negative affect, *p*s < .01) contexts, these trends were stronger in the homework context. The effect of parents’ self‐efficacy on parents’ autonomy support did not vary by context, *t*(1109) = 0.85, *p* = .40, but the effect on their control did, *t*(1019) = −2.55, *p* < .05. Although self‐efficacious parents used less control in both the homework (*β* = −.06, *p* < .05) and activity (*β* = −.14, *p* < .01) contexts, this trend was stronger in the activity context.

There were several effects of the demographic covariates on the learning context slopes for children’s engagement (see Table 3). The effects of parents’ educational attainment, child gender, and child grade on the frequency of child engagement varied by context, |*t*|s > 2.75, *p*s < .01: The higher parents’ educational attainment, the less often children engaged in math homework (*β* = −.18, *p* < .01), and the more often they engaged in math activities (*β* = .25, *p* < .001), such that math homework was more common than math activities in families where the participating parent had a college degree or less (*β*s < −.08, *p* < .05), but in families where parents had a more advanced degree, math homework was less common than math activities (*β* = .13, *p* < .001). Children in first grade engaged in math homework less often than did their counterparts in second grade (*β* = .11, *p* < .05), but the reverse was true for activities (*β* = −.22, *p* < .001), such that first graders engaged in less homework than activities (*β* = .10, *p* < .01), whereas second graders engaged in more homework than activities (*β* = −.23, *p* < .001). Girls and boys were equally engaged in math homework (*β* = .02, *p* = .60), but girls (vs. boys) engaged in fewer math activities (*β* = −.12, *p* < .05), with girls engaging in more homework than activities (*β* = −.12, *p* < .01) and boys engaging equally in the two (*β* = .02, *p* = .54).

There were also effects of the demographic variables on the learning context slope for the qualitative aspects of parents’ involvement. The effect of parents’ educational attainment on their autonomy support varied by context, *t*(960) = −2.97, *p* < .01. The more educated parents were, the less autonomy‐supportive they were in the math activities (*β* = −.09, *p* < .05), but not homework (*β* = .02, *p* = .50) context, such that parents’ autonomy support did not differ in the two contexts among parents with a college degree or less (|*β|*s < .04, *p*s > .17), but was higher in the homework than activity context for parents with a more advanced degree (*β* = −.08, *p* < .001). Mothers’ and fathers’ positive affect varied by context, *t*(665) = 4.02, *p* < .001. Mothers (*β* = .15, *p* < .001), but not fathers (*β* = −.02, *p* = .60), had more positive affect in the activity than homework context such that in the activity context mothers (vs. fathers) had more positive affect (*β* = .10, *p* < .05), but there was no difference between the two in the homework context (*β* = −.07, *p* = .13).

### Predicting children’s math motivation and achievement

To examine whether the quality of parents’ involvement contributes to children’s math motivation and achievement, we conducted multiple regression analyses using the *lavaan* package (Rosseel, 2012) in R (v 3.1.2) to handle missing data with the full information maximum likelihood method to reduce response bias (Duncan et al., 2006). Using this package, the ratio of each parameter estimate to its standard error is distributed as a *z* statistic. Separate multiple regression analyses were conducted for the math homework and activity contexts. Children’s math liking, challenge preference, and achievement at Wave 2 were each predicted from their math adjustment at Wave 1, parents’ educational attainment, parents’ gender, children’s gender, and children’s grade in school, along with the number of days for which parents completed the daily survey (Step 1). The frequency of children’s math engagement and parents’ involvement in the learning context at Wave 1 were added at Step 2. The four qualitative aspects of parents’ involvement (i.e., autonomy support, control, positive affect, and negative affect) at Wave 1 were included in Step 3.

As shown in Table 5, the only consistently significant predictor to emerge in terms of the qualitative aspects of parents’ involvement in children’s math learning was parents’ affectively negative involvement. The more parents’ involvement was affectively negative in the homework context, the lower children’s math liking, preference for challenge, and achievement 1 year later over and above their earlier math liking, preference for challenge, and achievement, *z*s < −2.71, *p*s < .01. Although not significant in the activity context, the effects were similar to those in the homework context, *z*s < −1.37, *p*s > .08. To optimize power and reliability, we examined parents’ involvement across the two math learning contexts. To this end, we took the mean across not only days as in the earlier analyses, but also the homework and activity contexts. Given that 90% of parents were involved in homework or activities at least once, this yielded a dataset with less missing data. Moreover, taking the mean across the two contexts allowed us to use assessments from more days yielding a more reliable predictor based on more instances. Parents’ affectively negative involvement was again the only consistent predictor of children’s math liking, preference for challenge, and achievement 1 year later over and above their earlier math adjustment (see Table 5), *z*s > 2.54, *p*s < .05.

**TABLE 5:** Multiple regressions predicting child math motivation and achievement over time

Predictors (Wave 1)	Math liking			Preference for math challenge			Math achievement		
	(Wave 2)			(Wave 2)			(Wave 2)		
	Homework	Activity	Combined	Homework	Activity	Combined	Homework	Activity	Combined
	*β*	*β*	*β*	*β*	*β*	*β*	*β*	*β*	*β*
Step 1									
Child math adjustment	.41***	.41***	.41***	.37***	.37***	.37***	.82***	.83***	.82***
Parent education	.00	.02	.01	.08	.07	.07	.09**	.09***	.09**
Parent gender	−.04	−.05	−.04	−.08	−.08	−.07	−.01	−.03	−.01
Child gender	−.05	−.05	−.05	−.01	−.02	−.02	.02	.01	.01
Child grade	−.10*	−.10*	−.11*	.03	.04	.03	−.10***	−.10***	−.10***
Number of daily reports	.06	.06	.08	.05	.06	.07	.04	.05	.05
Step 2									
Child engagement	.02	.06	.06	−.03	.11*	.05	.00	.04	.03
Parent involvement	−.03	−.07	.00	.02	−.02	.04	−.02	−.10***	−.07*
Step 3									
Parent autonomy support	.07	.04	.09	−.06	−.02	−.04	.01	.03	.01
Parent control	.09	.04	.08	−.01	−.01	−.01	−.03	.01	−.03
Parent positive affect	−.12*	−.02	−.11*	−.02	.01	.01	−.03	.00	−.01
Parent negative affect	−.20***	−.11	−.16**	−.17**	−.11	−.18**	−.11**	−.05	−.09*

Note

For the combined analyses, we took the mean across homework and activities. For parents’ education, −1 = less than a bachelor's degree, 0 = a bachelor's degree or equivalent, and 1 = an advanced graduate degree; for parent gender, −1 = fathers, 1 = mothers; for child gender, −1 = boys and 1 = girls; for child grade, −1 = first grade and 1 = second grade.

* *p* < .05

** *p* < .01

*** *p* < .001.

It is possible that the daily composites have a substantial error if parents were rarely involved over the 12‐day report period. For example, if parents were only involved once during the 12‐day period, the affective nature of their involvement may not reflect the general nature of their involvement but rather their unique state on that particular day. To ensure this was not the case, we conducted multiple regression analyses excluding the families in which parents provided fewer than two reports of each qualitative aspect of their involvement. As shown in Supporting Information (see Table S2), despite substantially smaller sample sizes, the results from this set of analyses for the effects of parents’ negative affect were quite similar to those reported including families with fewer than two reports of each qualitative aspect of their involvement (see Table 5). The only exception was that the effect of parents’ negative affect in the homework context on children’s preference for challenge was no longer significant, *z* = −1.89, *p* = .059. These analyses did not reveal any consistent effects of the other qualitative aspects of parents’ involvement, although parents’ control in the homework context predicted less preference for math challenge among children over time, *z* = −2.26, *p* < .05.

## DISCUSSION

Once children enter school, both math homework and activities provide a context for parents to support children’s math learning. However, relatively little is known as to whether one of these learning contexts may be more effective in fostering constructive involvement in children’s math learning among parents. The current research revealed that during early elementary school, parents’ involvement in children’s math homework tends to be affectively less positive and more negative than their involvement in children’s math activities. Notably, this tendency was mitigated to some extent when parents felt more efficacious in supporting children’s learning, such that the more efficacious parents felt the more positive and less negative their affect in both learning contexts, with this effect of feelings of efficacy being stronger in the homework (vs. activity) context. Parents’ affectively negative involvement, particularly in the homework context, was predictive over time of children’s dampened math liking, preference for challenging math, and math achievement. Taken together, these findings suggest that math homework may elicit less constructive involvement among parents than do math activities, with parents’ feelings of efficacy in helping children with math serving as a resource to optimize their involvement in both contexts, but particularly homework.

### Parents’ involvement in math homework and activities

When parents feel pressure or children have difficulties with schoolwork, parents are less constructively involved in children’s learning during elementary and middle school (e.g., Grolnick et al., 2002; Silinskas et al., 2015). Because math homework may be characterized more by pressure and difficulty than math activities, it was expected that parents’ involvement would be less autonomy‐supportive (vs. controlling) and affectively positive (vs. negative) in the homework (vs. activity) context. Indeed, parents were less affectively positive and more affectively negative when they were involved in math homework than activities, with the size of this difference falling in the medium range. Interestingly, the more educated parents were the less affectively positive their involvement, regardless of the context. This could be due to the higher pressure they feel for children to do well in math. Surprisingly, although parents’ control did not differ in the two contexts, their autonomy support was *higher* in the math homework (vs. activity) context, with this difference falling in the small range. It is possible that because we adapted the autonomy support and control items from measures originally designed for schoolwork (Cheung et al., 2016), these items were less relevant to math activities, particularly for autonomy support. For example, one of the autonomy support items asked about allowing children to take the lead or solve problems on their own, which is likely more relevant to homework than activities for which there may not be problems to solve.

Although the affective quality of parents’ involvement differed in the math homework and activity context, the frequency of parents’ involvement in these two contexts was similar on average. Children’s relative engagement in the two learning contexts, however, depended on children’s grade in school. During first grade, children were more often engaged in math activities than homework; but during second grade, they were more often engaged in math homework (vs. activities). Thus, it appears that as children receive more math homework, they compensate by decreasing their engagement in math activities. This trend of greater engagement in math homework than activities is likely to continue and even widen as children get older given the increase in homework over the school years (Snyder et al., 2019). If this is the case, as children get older, the math learning context may be one in which parents’ involvement is affectively less positive and more negative.

### Parents’ self‐efficacy as a resource for parents’ involvement

Consistent with the notion that parents’ feelings of efficacy in supporting children’s learning serve as a resource in their involvement in children’s learning (Hoover‐Dempsey & Sandler, 1997), parents’ feelings of efficacy were associated with their more frequent involvement in children’s math learning in both the homework and activity contexts as in prior research on parents’ involvement in general (e.g., Green et al., 2007; Hoover‐Dempsey et al., 1992) and in math specifically (e.g., Keating et al., 2021; O’Sullivan et al., 2014). Extending these findings, in the current research, the more efficacious parents felt, the more autonomy‐supportive and affectively positive and the less controlling and affectively negative their involvement. These effects were not trivial in size as they fell into the medium to large range. Although this trend was evident for both math homework and activities, parents’ self‐efficacy appeared to make a bigger difference for parents’ affect in the homework context where it was more strongly associated with heightened positive and dampened negative affect among parents than in the activity context. This may reflect a tendency for parents’ self‐efficacy to be particularly helpful in regulating their affect in the face of challenge as they feel more confident that they can assist children through the difficulties they encounter.

Given that parents’ feelings of efficacy in supporting children’s math learning are linked to the qualitative aspects of their involvement in children’s math learning, such feelings may be an important target for interventions to enhance parents’ involvement. However, a better understanding of what underlies parents’ feelings of efficacy is needed. For example, do such feelings reflect parents’ *confidence* in supporting children’s math learning as originally argued by Hoover‐Dempsey and Sandler (1997)? Or do they reflect parents’ *actual* or *perceived* math skills? It was not possible to comprehensively address this question directly in the current research as an assessment of parents’ math skills was not administered. However, parents’ educational attainment was included as a covariate in the analyses. Although positively associated with parents’ feelings of efficacy (see Table 2), parents’ educational attainment did not account for any of the self‐efficacy effects, which may have been the case if parents’ actual math skills underlie their feelings of efficacy. Parents also reported on their competence in math (i.e., “How comfortable are you with using math?”), which was associated with parents’ feelings of efficacy (*r* = .51, *p* < .01), but when added to the analyses we reported, did not account for the effects of parents’ feelings of efficacy in supporting children’s math learning. Thus, it is likely that the effects of parents’ feelings of efficacy documented here are due largely to their confidence in being able to effectively help children with math. However, in the later years of school when math is more complex, parents’ feelings of efficacy in supporting children’s math learning may only be useful if accompanied by advanced math skills.

### Implications of parents’ involvement for children’s math motivation and achievement

Although prior research has examined the link between qualitative aspects of parents’ involvement in children’s math homework and children’s math motivation and achievement (e.g., Doctoroff & Arnold, 2017; Silinskas et al., 2015), this is the first study to examine qualitative aspects of parents’ involvement in both children’s math homework and activities with attention to how they predict children’s math motivation and achievement over time adjusting for a variety of potential confounds, including children’s prior math motivation and achievement. Parents’ affectively negative involvement predicted children’s dampened math liking, preference for challenging math, and math achievement a year later. These effects fell in the small range, but may accumulate over time to undermine children’s math motivation and achievement. In addition, the effects only reached significance in the homework context, although they were in the same direction in the activity context. It may be that parents’ negative affect was generally not high enough in the math activity context to undermine children’s motivation and achievement. Another possibility is that an absence of negative affect does not facilitate children’s math motivation and achievement unless parents’ involvement supports relevant cognitive abilities among children, which may generally be the case in the homework but not activity context. The inverse effect for parents’ positive affect was not evident. In fact, parents’ heightened positive affect in the homework context predicted dampened math liking. Although it is unclear how much to make of this effect given that it was not evident across the indicators of children’s motivation and achievement and was not significant in the analyses in which parents had to have at least two reports of each qualitative aspect of parents’ involvement, it is possible that parents displayed positive affect to motivate children, which children did not find genuine, and even experienced as controlling. It also may be that heightened positive affect is viewed by children as a lack of empathy on the part of parents when children are struggling, which can undermine children’s motivation (Chen et al., 2018).

Neither parents’ autonomy‐supportive nor controlling involvement in children’s math homework or activities predicted children’s motivation or achievement. This was surprising given that autonomy‐supportive and controlling parenting predict children’s motivation and achievement in general over time (for a review, see Pomerantz et al., 2012). Moreover, research with children in elementary school focusing on parents’ involvement in math homework finds a concurrent association between more controlling involvement and poorer motivation and achievement (Silinskas & Kikas, 2019a; Silinskas et al., 2015). Although there were similar associations in the current research (see Table 2), once we took into account children’s prior math adjustment and other potential confounds, the associations were no longer evident. Across the analyses, the findings for parents’ autonomy support and control were often not in line with our hypotheses or prior research. It may be that parents’ autonomy‐supportive and controlling involvement operate differently for children’s math learning than for other types of learning. For challenging learning situations like math homework and activities, adopting autonomy‐supportive practices such as encouraging children to solve difficult math problems on their own may be ineffective if it leads to too much frustration when children cannot solve the problems relatively quickly. In addition, in the math learning context, controlling parenting may include instruction and scaffolding that facilitate children’s math skill development which may balance out the negative effects of parents’ control. It is also possible that the measurement of autonomy‐supportive and controlling practices involves more instances of involvement than can be captured in the 12‐day timeframe of the daily reports used in the current research.

### Limitations and future directions

Despite key methodological strengths, including the use of daily reports to assess parents’ involvement in two math learning contexts and assessment of both children’s math motivation and achievement nested with a longitudinal design, there are several limitations of the current research that merit consideration and suggest important future directions. First, although the multilevel analytic approach we used ruled out the possibility that differences in the nature of parents’ involvement in math homework and activities are due to differences between families, causal conclusions must be made with caution as differences from day to day within families may drive the differences. For example, the tendency for parents’ involvement in math homework to be more affectively negative than their involvement in math activities may not reflect the different characteristics of the two learning contexts but rather a tendency for parents to get involved in math homework, but not activities, even when they are in a bad mood as only children’s homework has a deadline and evaluation. It was also the case that the association between parents’ self‐efficacy in supporting children’s math learning and their involvement in children’s math homework and activities was concurrent. Consequently, although it appears likely that parents’ feelings of efficacy may serve as a resource for optimal involvement among parents, it is possible that the more parents engage in constructive involvement, the more they see themselves as effective in supporting children’s math learning. However, identifying the direction of effects between parents’ self‐efficacy and their involvement is challenging because parents’ self‐efficacy in helping with math may set the stage early on for their involvement, forming a stable system by the time children enter elementary school. Thus, an experimental approach manipulating parents’ self‐efficacy in helping with math to detect its influence on their involvement in children’s math learning may be fruitful (for a similar approach to identifying the causal role of parents’ beliefs, see Moorman & Pomerantz, 2010).

Second, the representativeness of the current sample is limited along several lines. For one, only a third of parents in the sample had less than a college degree. The sample was also comprised predominantly of white families residing in the Midwest with the majority of participating parents being mothers. Although there are similarities in parents’ involvement in children’s learning across parents’ educational attainment, race and ethnicity, and gender, there are also differences (e.g., Barger et al., 2019; Kim & Hill, 2015; Sibley & Dearing, 2014). This may be particularly true for math as it is often viewed as driven by natural intelligence (vs. hard work), which women and people of color may be viewed as lacking (e.g., Leslie et al., 2015). These kinds of views may contribute to parents’ feelings of efficacy in supporting children as well as the quality of their involvement in math learning. Interestingly, in the current research, mothers had more positive affect than did fathers in the activity, but not homework, context, suggesting that mothers’ involvement may be particularly constructive around math activities. This finding, however, should be interpreted with caution given the small number of fathers in the sample (i.e., only 20% of the sample). We also did not find ethnic or racial differences, but we had limited power to detect such differences given that the majority (i.e., 67%) of our sample was white. In addition, our analyses on parents’ involvement excluded parents who did not report being involved; thus, we cannot capture the contribution of such parents to children’s math motivation and achievement. In addition, it is possible that by excluding these parents we were not able to fully capture the qualitative aspects of parents’ involvement. For example, it may be that giving children full reign over their math homework and activities is a form of autonomy support—at least when children can handle such independence effectively.

Third, the daily reports we used to assess parents’ involvement in children’s math homework have a number of strengths, but they are susceptible to various reporting biases. For example, parents reported their involvement in a favorable light (e.g., more affectively positive than negative). Although this is in line with observations in the lab of parents’ affect when working with children on an academic activity (e.g., Else‐Quest et al., 2008; Moorman & Pomerantz, 2010), it is possible that self‐presentational biases suppress unconstructive biases in both daily reports and lab observations, which may have been one reason we did not find more effects of the quality of parents’ involvement on children’s math adjustment. Future work using a multi‐method approach to assessing parents’ involvement may lead to a more accurate portrait of their involvement in children’s math activities and homework and the predictive significance of such involvement. Although we made every effort to provide parents with a comprehensive set of examples of math activities, we may not have been comprehensive enough. For example, given that spatial abilities appear to contribute to math achievement (e.g., Ribeiro et al., 2020), this may have been an important type of activity to include. In addition, we did not distinguish between different types (e.g., constrained and unconstrained) of math activities in terms of their support of relevant cognitive skills (e.g., McCormick et al., 2020). This will be an important direction for future research, as will comparing parents’ involvement in children's math learning with their involvement in other domains of children's learning such as literacy to identify what is and is not specific to math in regard to the patterns identified in the current research.

## CONCLUSION

The current research makes inroads into understanding the quality of parents’ involvement in two common math learning contexts—homework and activities—during early elementary school. The findings suggest that math homework may be a less constructive context than math activities for parents to be involved in children’s learning as parents are affectively less positive and more negative in the math homework (vs. activity) context. Importantly, parents’ negative affect, particularly in the math homework context, predicted children’s math motivation and achievement over time. Parents’ feelings of efficacy in supporting children’s math learning were linked to more constructive involvement in both math homework and activities, appearing to be particularly likely to help parents tamp down negative affect when assisting with homework (vs. activities). Thus, interventions aimed at increasing parents’ feelings of efficacy in supporting children’s math learning may be useful in optimizing parents’ contribution to children’s math motivation and achievement in early elementary school.

## Supporting information

Supplementary Material
