# Supplementary material for: Parents’ daily involvement in children’s math homework and activities during early elementary school
Source: Child Dev. 2022 Apr 18;93(5):1347–64. doi: 10.1111/cdev.13774 (PMC9542134; doi:10.1111/cdev.13774)
Supplement: Supplementary file 1 — Supplementary Material [file CDEV-93-1347-s001.docx]

**Parents’ Daily Involvement in Children’s Math Homework and Activities**

**During Early Elementary School**

**Online Supplemental Materials**

**Table S1**

*Descriptives for the Frequency of Child Engagement in Math Activities*

|  | *M* | *SD* |
| --- | --- | --- |
| 1. Math flashcards, workbooks, or worksheets | 0.08 | 0.16 |
| 2. Math quizzing | 0.05 | 0.14 |
| 3. Everyday math | 0.14 | 0.20 |
| 4. Math card games, dice games, or board games | 0.05 | 0.13 |
| 5. Math games on a digital device | 0.06 | 0.14 |
| 6. Other activities | 0.03 | 0.08 |
| Total | 0.26 | 0.26 |
| *Notes*. There were 483 parent-child dyads who provided reports for the math activities. Means presented here can be interpreted as the proportion of days children engaged in each type of activity. Parents were given a list of math activities adapted from those used in measures designed for parents of children who had not yet entered school (e.g., LeFevre et al., 2009; Skwarchuk et al., 2014; Zippert & Rittle-Johnson, 2020): (1) *math flashcards, workbooks, or worksheets* in paper form or on a digital device (e.g., a tablet or computer); (2) *math quizzing* (e.g., gave child addition problems or math word problems); (3) *everyday math* (e.g., measuring things while cooking or telling time); (4) *math card games, dice games, or board games*; (5) *math games on a digital device* (e.g., a tablet or computer); (6) *other*. | | |

**Table S2**

*Multiple Regressions Predicting Child Math Motivation and Achievement Over Time Among Families with at Least Two Reports of each Qualitative Aspect of Parent Involvement*

|  | Math Liking | | | Preference for Math Challenge | | | Math Achievement | | |
| --- | --- | --- | --- | --- | --- | --- | --- | --- | --- |
|  | (Wave 2) | | | (Wave 2) | | | (Wave 2) | | |
| Predictors | *Homework* | *Activity* | *Combined* | *Homework* | *Activity* | *Combined* | *Homework* | *Activity* | *Combined* |
| (Wave 1) | β | β | β | β | β | β | β | β | β |
| Step 1 |  |  |  |  |  |  |  |  |  |
| Child math adjustment | 0.47^***^ | 0.39^***^ | 0.45^***^ | 0.38^***^ | 0.35^***^ | 0.37^***^ | 0.81^***^ | 0.82^***^ | 0.81^***^ |
| Parent education | 0.00 | -0.03 | 0.01 | 0.05 | 0.14^*^ | 0.11^*^ | 0.10^**^ | 0.12^**^ | 0.09^**^ |
| Parent gender | -0.02 | -0.01 | -0.01 | -0.10 | -0.12 | -0.09 | -0.03 | -0.05 | -0.04 |
| Child gender | -0.01 | 0.00 | -0.06^*^ | -0.02 | -0.06 | -0.05 | 0.01 | 0.02 | 0.01 |
| Child grade | -0.17^**^ | -0.02 | -0.11^*^ | 0.01 | 0.09 | 0.01 | -0.07 | -0.08 | -0.08^*^ |
| Number of daily reports | 0.11 | 0.18^*^ | 0.15 | -0.11 | -0.03 | -0.02 | 0.01 | 0.09^*^ | 0.03 |
| Step 2 |  |  |  |  |  |  |  |  |  |
| Child engagement | -0.04 | 0.15^*^ | 0.10 | -0.14^*^ | 0.11 | 0.03 | 0.01 | 0.04 | 0.04 |
| Parent involvement | -0.05 | 0.02 | -0.04 | -0.10 | -0.06 | -0.07 | -0.02 | -0.09^*^ | -0.09^**^ |
| Step 3 |  |  |  |  |  |  |  |  |  |
| Parent autonomy support | 0.05 | 0.13 | 0.07 | -0.10 | 0.01 | -0.01 | 0.01 | 0.02 | 0.04 |
| Parent control | 0.11 | 0.08 | 0.07 | -0.15^*^ | 0.02 | -0.05 | -0.02 | 0.03 | 0.00 |
| Parent positive affect | -0.07 | -0.07 | -0.07 | 0.02 | -0.04 | 0.01 | 0.00 | -0.05 | -0.02 |
| Parent negative affect | -0.14^*^ | -0.07 | -0.11^*^ | -0.13 | -0.17 | -0.18^**^ | -0.10^*^ | -0.01 | -0.10^**^ |

*Note*. Only families who provided at least two reports of each qualitative aspect of parent involvement for the context included in the analysis were included in the analysis. Thus, 259 families were included in the analyses evaluating the homework context, 205 in the analyses evaluating the activity context, and 377 in the analyses evaluating the two contexts combined. For parents’ education, -1 = less than a bachelor’s degree, 0 = a bachelor’s degree or equivalent, and 1 = an advanced graduate degree; for parent gender, -1 = fathers, 1 = mothers; for child gender, -1 = boys and 1 = girls; for child grade, -1 = 1^st^ grade and 1 = 2^nd^ grade.

^*^*p* < .05, ^**^*p* < .01, ^***^*p* < .001.

**Figure S1**

*Daily Reports Flow Chart*

**

**Equations for MLMs Comparing the Math Homework and Activity Context**

The following Level 1 (context-level) function (1) was specified to describe the data from each day for each family:

$Y_{ijk}=A_{ij}+B_{ij}\left( Context \right)_{ijk}+e_{ijk}$ (1)

The frequency of child engagement and parents’ involvement as well as the quality of parents’ involvement for a given context (k) at a particular day (j) for a particular family (i) were modeled by the intercept (*A_ij_*) and the variance for each learning context, such that $B_{ij}$ represents the difference between the math homework and activity contexts. The error term ($e_{ijk}$) represents unexplained variance. The Level 1 equation (1) allowed for the examination of child and parent variables as a function of the two math learning contexts. To this end, the two learning contexts, were contrast coded with math homework coded as -1 and math activity coded as 1, so that the intercept represents the mean across the two learning contexts.

$A_{ij}=A_{i}+a_{ij}$ (2)

$B_{ij}=B_{i}+b_{ij}$ (3)

We added a Level 2 (day-level) function (2 and 3) to account for the potential fluctuations across days. The intercept $A_{ij}$ and slope $B_{ij}$for Level 1 (context-level; equation 1) were broken down to $A_{i}$, which represents the mean intercepts for a particular family (i) across days and variance for a particular day ($a_{ij}$), $B_{i}$, which represents the mean slop (i.e., subject difference) for a particular family (i) across days and variance for a particular day ($b_{ij}$).

$A_{i}=\alpha+\alpha_{1}\left( Parent education \right)_{i}+\alpha_{2}\left( Child Gender \right)_{i}+\alpha_{3}\left( Child Grade \right)_{i}+\alpha_{4}\left( Parent Efficacy \right)_{i}+a_{i}$ (4)

$B_{i}=\beta+\beta_{1}\left( Parent education \right)_{i}+\beta_{2}\left( Child Gender \right)_{i}+\beta_{3}\left( Child Grade \right)_{i}+\beta_{4}\left( Parent Efficacy \right)_{i}+b_{i}$ (5)

To examine whether parents’ feelings of efficacy and demographic covariates contribute to the variability in children’s engagement and parents’ involvement in the math homework and activity context, Level 3 (between-dyad) equations (4 and 5) were included. In these equations, the intercept ($A_{i}$)—that is, the mean values of child or parent measures across contexts—and slope ($B_{i}$)— that is, the association between math homework and math activity context—were predicted from parents’ feelings of efficacy and demographic covariates. Error terms contributing to unexplained variance are represented by $a_{i}$ and $b_{i}$.

References

LeFevre, J.-A., Skwarchuk, S.-L., Smith-Chant, B. L., Fast, L., Kamawar, D., & Bisanz, J. (2009). Home numeracy experiences and children’s math performance in the early school years. *Canadian Journal of Behavioural Science/Revue Canadienne Des Sciences Du Comportement*, *41*(2), 55–66. https://doi.org/10.1037/a0014532

Skwarchuk, S.-L., Sowinski, C., & LeFevre, J.-A. (2014). Formal and informal home learning activities in relation to children’s early numeracy and literacy skills: The development of a home numeracy model. *Journal of Experimental Child Psychology*, *121*, 63–84. https://doi.org/10.1016/j.jecp.2013.11.006

Zippert, E. L., & Rittle-Johnson, B. (2020). The home math environment: More than numeracy. *Early Childhood Research Quarterly*, *50*, 4–15. https://doi.org/10.1016/j.ecresq.2018.07.009
